# Effect of mass paediatric influenza vaccination on existing influenza vaccination programmes in England and Wales: a modelling and cost-effectiveness analysis

**DOI:** 10.1016/S2468-2667(16)30044-5

**Published:** 2017-02

**Authors:** David Hodgson, Marc Baguelin, Edwin van Leeuwen, Jasmina Panovska-Griffiths, Mary Ramsay, Richard Pebody, Katherine E Atkins

**Affiliations:** a Centre for Mathematics, Physics and Engineering in the Life Sciences and Experimental Biology, University College London, London, UK; b Clinical and Operational Research Unit, Department of Mathematics, University College London, London, UK; c Faculty of Epidemiology and Population Health, London School of Hygiene & Tropical Medicine, London, UK; d Department of Global Health and Development, London School of Hygiene & Tropical Medicine, London, UK; e Respiratory Diseases Department, Public Health England, London, UK; f Immunisation, Hepatitis and Blood Safety Department, Public Health England, London, UK; g Department of Infectious Disease Epidemiology, Medical Research Council Centre for Outbreak Analyses and Modelling, School of Public Health, Imperial College London, London, UK

## Abstract

**Background:**

In 2013 England and Wales began to fund a live attenuated influenza vaccine programme for individuals aged 2–16 years. Mathematical modelling predicts substantial beneficial herd effects for the entire population as a result of reduced influenza transmission. With a decreased influenza-associated disease burden, existing immunisation programmes might be less cost-effective. The aim of this study was to assess the epidemiological effect and cost-effectiveness of the existing elderly and risk group vaccination programme under the new policy of mass paediatric vaccination in England.

**Methods:**

For this cost-effectiveness analysis, we used a transmission model of seasonal influenza calibrated to 14 seasons of weekly consultation and virology data in England and Wales. We combined this model with an economic evaluation to calculate the incremental cost-effectiveness ratios, measured in cost per quality-adjusted life-years (QALY) gained.

**Findings:**

Our results suggest that well timed administration of paediatric vaccination would reduce the number of low-risk elderly influenza cases to a greater extent than would vaccination of the low-risk elderly themselves if the elderly uptake is achieved more slowly. Although high-risk vaccination remains cost-effective, substantial uncertainty exists as to whether low-risk elderly vaccination remains cost-effective, driven by the choice of cost-effectiveness threshold. Under base case assumptions and a cost-effectiveness threshold of £15 000 per QALY, the low-risk elderly seasonal vaccination programme will cease to be cost-effective with a mean incremental cost-effectiveness ratio of £22 000 per QALY and a probability of cost-effectiveness of 20%. However, under a £30 000 per QALY threshold, the programme will remain cost-effective with 83% probability.

**Interpretation:**

With the likely move to decreased cost-effectiveness thresholds, reassessment of existing risk group-based vaccine programme cost-effectiveness in the presence of the paediatric vaccination programme is needed.

**Funding:**

National Institute for Health Research, the Medical Research Council.

## Introduction

Individuals at high risk of serious complication after an influenza infection have historically been the target for seasonal influenza annual vaccination programmes worldwide.^1^, ^2^, ^3^ However, because of the indirect effects of vaccination, termed herd protection, the vaccination of groups who are important for transmission of infection is often also cost-effective.^4^, ^5^ A large proportion of this transmission is attributable to children and adolescents because of the relatively high number of contacts they have with others,^6^, ^7^, ^8^ the fraction of these contacts that involve touching,^6^, ^7^, ^8^ and their susceptibility to influenza infection.^9^, ^10^ Some countries have broadened their seasonal influenza vaccine recommendations to immunise healthy children and adolescents every year, for example in the USA^11^ and more recently in the UK in 2012.^12^, ^13^

If paediatric immunisation programmes gain high coverage early enough in the influenza season to interrupt transmission to risk groups, any existing risk group-based vaccine programme becomes less cost-effective. If a national paediatric programme renders elderly and risk group vaccination programmes not cost-effective, then removing annual vaccination from these target groups will allow a large annual saving. Previous analysis in the context of the UK suggests that substantial uncertainty exists regarding the cost-effectiveness of an elderly low-risk programme in the presence of a moderate vaccine uptake of 50% in healthy children^12^, ^13^ and adolescents, with a third of simulations finding the elderly vaccine not to be cost-effective.

To reduce seasonal influenza-associated serious disease in elderly people and in individuals clinically at risk from influenza-related disease, England and Wales are introducing a publicly funded universal funded paediatric vaccination programme,^12^, ^13^ using live attenuated influenza vaccine (LAIV). The LAIV programme began in 2013 and offered the intranasal vaccines to all healthy children aged 2–3 years and to healthy children aged 4 years and those in primary grades 1–6 (children commencing the school year aged 5–10 years) who were enrolled in a pilot study done in seven regions. In 2014–15, the programme extended to all children aged 2–4 years, and the ongoing pilot studies expanded to include some regions offering vaccines to secondary school-aged adolescents in school grades 7–8 (those commencing the school year aged 11–12 years). In 2015–16, the programme offered vaccines to primary grades 1–2 (those commencing the school year aged 5–6 years) and continued to offer vaccines to children aged 2–4 years and children in the previous pilot areas. From 2016 onwards, the programme extends year on year to children in the rest of the primary school grades (aged 5–10 years when commencing the school year) and secondary school adolescents (aged 11–15 years when commencing the school year).^14^ Case-control studies have been implemented over this period to ascertain the direct effectiveness across each season.^15^, ^16^

**Research in context**

**Evidence before this study**

Using a transmission model, our previous study showed that a paediatric vaccination programme can effectively control seasonal influenza because of the substantial herd protection conferred to unvaccinated children and adults. The decision to implement a paediatric vaccine programme in England was initiated after the evidence presented in a later study that combined the previous mathematical model with a cost-effectiveness analysis. Although this study showed paediatric vaccination to be very cost-effective, the study only superficially assessed the effect of this programme on existing target group vaccination.

**Added value of this study**

In this Article, we assess the epidemiological effect and cost-effectiveness of the existing elderly and high-risk group vaccination in the presence of paediatric vaccination. Our study suggests that a mass paediatric vaccination programme will not affect the existing recommendation of risk group vaccination in England. However, the continued cost-effectiveness of a low-risk elderly vaccination programme is uncertain.

**Implications of all the available evidence**

This study highlights the importance of a cost-effective paediatric vaccination scheme in curtailing influenza transmission to the most vulnerable risk groups. However, important uncertainties exist surrounding the cost-effectiveness of elderly programmes in the UK that might warrant reconsideration of this strategy in the coming years. We suggest that other countries introducing paediatric vaccination should reconsider their full vaccination schedule in view of these results.

Using England as a case study, we assess the likely effect and cost-effectiveness of the existing elderly and risk group vaccination programme under the new policy of mass paediatric vaccination. Specifically, we assess whether maintaining a low-risk elderly and high-risk seasonal influenza vaccination programme remains cost-effective in the presence of preschool and school-based vaccination with varying vaccine coverages achieved at different speeds. Although the focus of our study assesses the situation after the decision in England in 2012 to introduce preschool-age and school-age influenza vaccination, we extend this result to general policy considerations for other countries where paediatric programmes are currently under review.

## Methods

### Epidemiological effect of paediatric vaccination

For this cost-effectiveness analysis, to predict the direct and indirect effects of seasonal influenza vaccine programmes, we use a previously described transmission model of seasonal influenza in England and Wales that is calibrated to 14 seasons of data.^12^, ^13^ Using this calibrated model, we assessed the influenza incidence in two groups: the high-risk population (individuals older than 6 months who have a diagnosed clinical disorder that puts them at risk of complications after influenza infection); and the low-risk elderly population (individuals older than 64 years who are not categorised as high risk). We assess the influenza incidence with and without low-risk elderly and high-risk vaccination, in the presence of three different paediatric programmes: (1) preschool age (2–4 years) only; (2) preschool and primary school age (2–10 years); and (3) preschool, primary, and secondary school age (2–16 years). Because children are known to be epidemiological drivers for seasonal influenza transmission, the speed at which children are vaccinated will probably affect the disease burden of the rest of the population. Therefore, we considered three administration speeds: (1) slow (uptake achieved between Jan 1 and Jan 31); (2) linear (uptake achieved between Oct 1 and Jan 31); and (3) fast (uptake achieved between Oct 1 and Oct 31). We also assessed the effect of different paediatric vaccine coverages on the effectiveness of the low-risk elderly and high-risk vaccine programmes.

### Transmission model and LAIV assumptions

We use an age-specific and risk-specific mathematical model that captures seasonal influenza transmission in England and Wales. The model is calibrated to the number of influenza-like illness consultations and the frequency of virological confirmations from 1995 to 2009. The calibrated model is parameterised with data on vaccination coverage, vaccine uptake speeds, and vaccine effectiveness data for elderly and high-risk inactivated influenza vaccination (IIV) in the absence of any paediatric vaccination. We calibrated the model using a Bayesian evidence synthesis approach, which captures uncertainty in the model parameters and is able to generate a distribution of model outcomes consistent with available data. The model captures the dynamics of A/H1N1, A/H3N2, and B strains separately. In each year, IIV is either matched or unmatched to the circulating strain. IIV efficacy was assumed to be 70% for people younger than 65 years and 46% for people aged 65 years or older for matched years, and 42% for people younger than 65 years and 28% for people aged 65 years or older for unmatched years. More details on the model and model parameters have been published previously.^12^

After the calibration, we used the model to predict the distribution of elderly and high-risk influenza cases averted in the presence of the new LAIV programme. In our base case scenario, we assumed LAIV efficacy across all strains was equal to IIV efficacy. Unless otherwise specified, our model was parameterised using the observed vaccine coverage and the speed of the uptake reported during the paediatric programme rollout for children aged 2–4 years and the pilot programme for children aged 4–16 years.^17^, ^18^

### Incremental cost-effectiveness of the elderly vaccination programme

Extending the economic framework previously used for decision making on the paediatric programme in England,^12^, ^13^ we assessed the net benefits accrued from continuing both the low-risk elderly and high-risk annual influenza vaccine programmes in England in the presence of a paediatric programme. We present the results in terms of the incremental cost for each quality-adjusted life-year gained (£ per QALY). For our base case analysis, we use 70% coverage for all children aged 2–16 years (consistent with uptakes in Scotland and Wales), together with the reported uptake speeds achieved in England for preschool-age children and school-age children (71% *vs* 40% achieved coverage by Oct 31, 89% *vs* 87% by Nov 30, and 94% *vs* 100% by Dec 31).^18^ For additional analyses, we also assessed the net benefits achieved under a range of feasible paediatric vaccination coverages (status quo, 50%, and 90%), together with a fast administration of children and adolescents by the end of October.

Officially, for an intervention to be deemed cost-effective in England, its incremental cost-effectiveness ratio (ICER) must typically have been less than £20 000 per QALY, although interventions with ICERs less than £30 000 per QALY were also considered.^19^ The mean ICER and the probability that the ICER falls below these thresholds (ie, the probability of cost-effectiveness) is also considered in the decision to fund a programme. Methodological research suggests that this threshold should be reduced to £13 000 per QALY^20^ and there is currently debate about whether the threshold will be updated.^21^ In practice, a threshold of about £15 000 per QALY would probably be deemed cost-effective. With these issues in mind, we present the mean ICER, and cumulative probabilities for three thresholds: £15 000 per QALY, £20 000 per QALY, and £30 000 per QALY.

### Economic evaluation assumptions

To calculate the cost-effectiveness of the elderly programme and high-risk programmes, we integrated the transmission model into an economic evaluation. The economic evaluation tracks the number of general practitioner consultations, hospital admissions, and deaths for each year for the three strains. QALYs lost are assumed for febrile cases to have a mean of 7·49 × 10^−3^, and for cases admitted to hospital, to be normally distributed with a mean of 0·018 (SD 0·0018).^13^ Vaccine costs associated with vaccine price reimbursement and administration were triangularly distributed (£11·00, £15·55, £20·00). General practitioner and hospital treatment costs are assumed to be normally distributed with mean prices £37·00 (SD 8·40) and £839·00 (192·10). Further details of the specific health economic values used, including the health burden of each of the associated health outcomes has been previously published.^13^ No discounting was applied because the economic evaluation results report the uncertainty in the cost per QALY gained over a single year.

### Statistical analysis

The analysis was run in R, using R Studio with the R package fluEvidenceSynthesis. Plots were drawn using Mathematica version 10.3.0.0.

### Role of the funding source

The funder of the study had no role in study design, data collection, data analysis, data interpretation, or writing of the report. KEA, MB, JP-G, EvL, RP, and MR had access to the raw data. The corresponding author had full access to all of the data in the study and the final responsibility for the decision to submit for publication.

## Results

**Figure 1:**
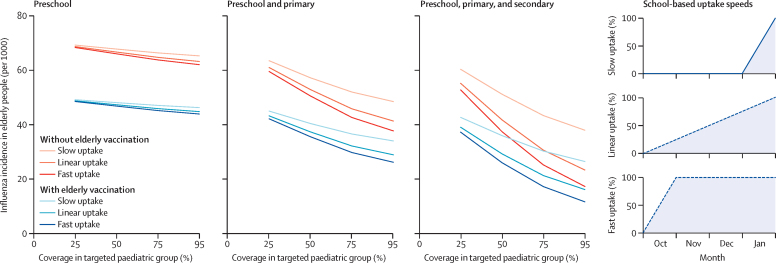
Effect of low-risk elderly and high-risk vaccination programmes in the presence of paediatric vaccination administered at different speeds through the influenza season Preschool are children aged 2–4 years, primary are children aged 5–10 years, and secondary are adolescents aged 11–16 years. The paediatric vaccination uptake speeds are associated with the accumulation of the fixed paediatric vaccine coverage across October, November, December, and January.

As a universal paediatric programme expands to older ages (ie, from preschool age [2–4 years] to primary age [children commencing the school year aged 5–10 years] to secondary age [adolescents commencing the school year aged 11–15 years]), the number of cases of influenza averted by an elderly or high-risk vaccination programme diminishes (figure 1). Importantly, however, as the paediatric programme expands, and there are concurrent drops in influenza incidence in the elderly and high-risk populations, the speed at which the paediatric programme is implemented becomes important. A rapid implementation each year of any vaccination programme of preschool, primary school, and secondary school children, even in the absence of an elderly vaccine programme, would avert a similar number of cases as the same paediatric programme implemented gradually over the influenza season in the presence of an elderly programme.

**Figure 2:**
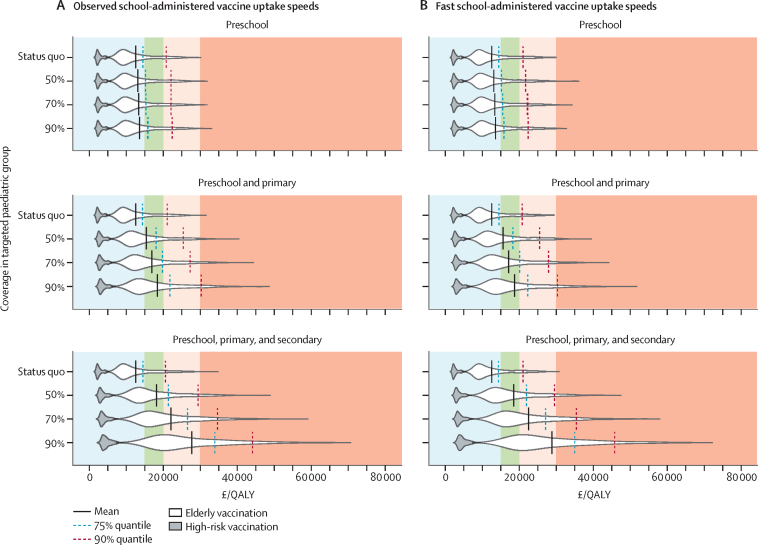
Cost-effectiveness of low-risk elderly and high-risk vaccination programmes in the presence of paediatric vaccination Preschool are children aged 2–4 years, primary are children aged 5–10 years, and secondary are adolescents aged 11–16 years. (A) All paediatric vaccination coverage is administered at speeds consistent with those reported during the full rollout of preschool or the pilot rollout of school-age children. (B) All paediatric vaccination coverage is administered by the end of October (fast uptake). Cost-effectiveness regions are coloured for readability: more than £30 000 per QALY (orange; not cost-effective), £20 000–30 000 per QALY (pink; cost-effective under current protocol), £15 000–20 000 per QALY (green; very cost-effective under current protocol), and less than £15 000 per QALY (blue; cost-effective under proposed protocol). The dark grey area corresponds to the incremental cost-effectiveness of a high-risk vaccine programme in the presence of the respective paediatric vaccine programme. The white areas correspond to the incremental cost-effectiveness of an elderly vaccine programme in the presence of the respective paediatric programme. Each grey and white probability distribution area is scaled to have a fixed maximum height, whereas each distribution represents an area of one unit. The mean (solid), 75% (dashed), and 90% (dotted) quantiles are shown to indicate in which cost-effectiveness region the mean of the distribution, 75%, or 90% of its simulations lie. QALY=quality-adjusted life-year.

Because of the high morbidity and mortality in the high-risk group, vaccination of these individuals remains cost-effective in the presence of all paediatric strategies with an ICER of less than £15 000 per QALY gained for all simulations (figure 2). By contrast, the presence of paediatric vaccination produces a qualitative shift in the ICER for low-risk elderly vaccination.

Under the 70% vaccine coverage base case, we find that although low-risk elderly vaccination is likely to maintain an ICER of less than £30 000 per QALY (83% of simulations), the ICER itself has increased by £9982 per QALY relative to no paediatric programme. Compared with no paediatric programme, the low-risk elderly programme has a much higher chance of falling between £20 000 per QALY and £30 000 per QALY (29%, compared with 12%) or more than £30 000 per QALY (17%, compared with 1%; figure 2). Conversely, with a cost-effectiveness threshold of £15 000 per QALY, the elderly programme is no longer cost-effective, with a mean ICER of £22 000 per QALY, and 80% of simulations providing ICER estimates of greater than £15 000 per QALY.

To assess the effect of paediatric uptake speed, we calculated the cost-effectiveness of elderly vaccination under a possible scenario in which the target coverage (70% vaccine uptake) was reached by the beginning of November (figure 2) compared with the present scenario, in which school-based administration increases gradually from October through December; achievement of the target coverage earlier (ie, by the beginning of November) resulted in a reduction in the cost-effectiveness of elderly vaccination and an increase in the ICER by £479 per QALY.

Our model suggests that changing the cost-effectiveness threshold across a reasonable range determines whether the low-risk elderly vaccination remains cost-effective. For example, even under optimistic conditions of fast delivery by the beginning of November and a coverage of 90% for the 2–16 years paediatric programme, the probability that elderly vaccination is cost-effective at £30 000 per QALY is still 68%. However, if the cost-effectiveness threshold falls to £15 000 per QALY under likely base case conditions, the elderly programme will cease to be cost-effective, with only 5% of simulations falling below the threshold (figure 2).

**Figure 3:**
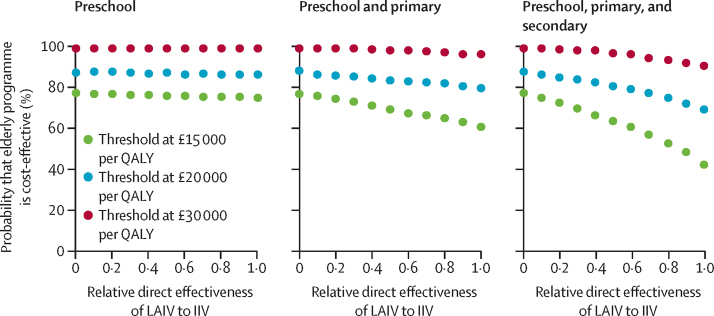
Effect of the whole-season direct effectiveness of live attenuated influenza vaccine on the probability that the elderly vaccination programme is cost-effective Preschool are children aged 2–4 years, primary are children aged 5–10 years, and secondary are adolescents aged 11–16 years. Paediatric vaccination coverage is set at 70% and is reached at speeds consistent with those observed during the full rollout of preschool and the pilot rollout of school-age children. Three different incremental cost-effectiveness ratio thresholds are considered: £30 000 per QALY (red; cost-effective), £20 000 per QALY (blue; very cost-effective under current protocol), and £15 000 per QALY (green; cost-effective under proposed protocol). A relative direct effectiveness of 1 corresponds to the LAIV having the same whole-season direct effectiveness as the IIV (base case value), and a relative direct effectiveness of 0 corresponds to the LAIV having no effect on influenza epidemiology and is therefore equivalent to the cost-effectiveness of the elderly programme with no paediatric coverage. LAIV=live attenuated influenza vaccine. QALY=quality-adjusted life-year. IIV=inactivated influenza vaccine.

We also assessed the effect of a reduced whole-season direct effectiveness of LAIV relative to the IIV that is currently given to elderly and high-risk individuals (figure 3). Under base case assumptions, the probability that the elderly programme is cost-effective increases as the whole-season LAIV direct effectiveness decreases. Under the conservative threshold of £15 000 per QALY, reducing LAIV effectiveness to half of IIV effectiveness increases the probability that the elderly programme is cost-effective from 42% to 65%.

## Discussion

We used a previously described calibrated mathematical model of seasonal influenza transmission in England and Wales to assess the likely epidemiological and economic effect of a paediatric programme, which is currently being rolled out across England, on the existing seasonal influenza vaccine programme. We found that under reasonable assumptions of vaccine coverage achieved in the pilot schemes, uncertainty exists about the continuing cost-effectiveness of the low-risk elderly vaccination programme that is available to all individuals with no underlying chronic disorders. The uncertainty surrounding these results stems primarily from the cost-effectiveness threshold assumed. We found that vaccinating high-risk individuals was always cost-effective.

Our analysis shows the potentially large effect of additional vaccination programmes on the cost-effectiveness of existing schemes. Particularly, the analysis highlights the importance of the assessment of existing influenza vaccination policies in the presence of increased herd protection after paediatric vaccination. Although our results suggest that vaccinating high-risk individuals will always be cost-effective over all plausible outcomes of the paediatric vaccination programme, the decision to maintain low-risk elderly vaccination is not so straightforward. As the new paediatric programme in England and Wales increases its scope from preschool-age children only to also include school-age children, with uptake consistent with other school-based programmes, significant uncertainty will probably arise concerning the cost-effectiveness of a low-risk elderly vaccination programme. Our analysis suggests three important questions for policy makers in countries wishing to introduce a paediatric programme (panel).

In England, the LAIV school-based pilot programme aimed at children aged 5–10 years reached a coverage of 52% in the first season of introduction and 57% in the second.^18^ However, national school-based administration of vaccines usually reaches much higher coverage. For example, at present, the first dose uptake for the human papillomavirus vaccine in England for girls aged 12–13 years is 89%,^22^ and for the 3-in-1 Tetanus-Diphtheria-Polio in Scotland for students aged 15–16 years is 88%.^22^, ^23^ Although the human papillomavirus and 3-in-1 vaccines are usually administered through the entire school year, influenza vaccines must achieve the target coverage in a short timeframe, typically before the Christmas holidays. As such, once the LAIV programme is established, a school-based administration of LAIV will probably achieve much higher coverage than the coverage that exists at present, but not as high as the coverage achieved by other school-administered vaccines. Unlike the timings for preschool, elderly, and high-risk vaccine administration, which are patient-led, the timing for a school-based vaccine administration would be determined by the regional National Health Service (NHS) commissioner, and is limited by the capacity of the providers, the number of schools, and the available days in the school term. If maximum uptakes are to be reached before November, additional resources would probably need to be diverted to enable sufficient local vaccination teams. Allocation of extra resources to regional NHS groups responsible for school-based vaccine administration might result in an increase in either the speed at which maximum coverage is met or the total vaccine uptake. Our results suggest that rapid administration of paediatric vaccines will provide substantial protection to both children and adults. This result is consistent with research^7^, ^8^ suggesting that influenza transmission is driven in most seasons by the young. In this analysis, we assess national vaccination policies; however, differences in vaccine uptake, speed of administration, and the population age distribution across NHS regions will probably result in heterogeneity in herd protection levels reached.

If the same number of LAIV doses were purchased, administration of them by the end of October would prevent the need for the entire low-risk elderly vaccine programme (figure 1). Because children and adolescents are known to be drivers of influenza transmission^6^, ^7^, ^8^ and, as such, are targeted for vaccination, cost-effective vaccination depends not only on the target population, but also on the speed at which the population is targeted.^24^

The US Advisory Committee on Immunization Practices temporarily withdrew their recommendation for LAIV administration on the basis of evidence from the USA for inefficacy of the vaccine, by contrast with other settings such as the UK.^25^ Calculation of the season-wide direct effectiveness of LAIV is problematic for several reasons: first, the direct effectiveness is known to change with age of recipient, number of vaccine doses (eg, two doses in the USA *vs* one in the UK), and endpoint measured (eg, virologically confirmed or syndromic presentation only); and second, seasonal fluctuations exist in the efficacy of the vaccine due to strain composition of the vaccine, frequency of circulating strains, and vaccine manufacture. Our sensitivity analysis assesses the likely effect of variation in the season-wide direct effectiveness. Because of the wide variation in empirical estimates for direct vaccine effectiveness, particularly between A and B strains, the extent to which LAIV efficacy differs from IIV remains unclear.

Quantifying the population-level effect of a paediatric vaccination programme relies on capturing the social mixing patterns that facilitate disease transmission. Although this study uses empirical data on contact patterns from the same time period as the clinical data to which the model is calibrated, the methodology has some important caveats. For example, how the risk of influenza infection varies with the number of social contacts relative to their type or duration (eg, familial relationships in the same household, workplace meetings, etc) is not generally not well understood; additionally, how behaviour changes as a result of infection, and the implications for disease transmission, are also uncertain. Through consideration of the empirical-derived contact patterns that are used in the model as prior information, which updates to reflect other sources of data during the model calibration procedure, the dependency of the model on these data can be reduced. The accuracy of the model predictions are also contingent on the disease burden data to which the model is calibrated. Although the modelling approach is able to convey uncertainty in the predictions, robust age-specific influenza surveillance information is key for accurate model predictions.

With substantial uncertainty around the cost-effectiveness of an elderly vaccination in the presence of a paediatric programme in England, other developed countries with a large fraction of the population older than 65 years might wish to reassess their funded elderly programme. In 2014, 9·5 million individuals were older than 65 years in England, 17·6% of the total population and the same number of those aged 2–16 years.^26^ Assuming an elderly vaccine uptake of 70% and a dose and administration cost of about £16 per vaccine, discontinuing elderly vaccination would save more than £106 million annually.

Most developed countries have an ageing population as a result of a decreasing birth rate and an increasing life expectancy. These demographic shifts will not only increase the scale of an age-targeted programme, but they will also affect their cost-effectiveness through changes in age-specific social mixing patterns that affect the level of herd protection accrued. Frequent reassessment of policy decisions is essential to ensure these changes are accounted for.

The evidence needed to withdraw a funded vaccine programme might be different to that needed to initiate one. Should a decision be under consideration to remove a long-standing vaccination programme as a result of improved public health measures elsewhere, it might be wise to offer a scaled-back programme rather than completely remove an established policy. How such a scaled-back programme would be implemented depends on the details of the at-risk groups. For example, 55% of the high-risk population are themselves older than 65 years. Alternatively, extenuating circumstances might exist that take precedent over low probabilities of cost-effectiveness. Moreover, our results suggest that in countries that currently have no funded influenza vaccination programme in place, it is necessary to consider a suite of age-group and risk-group based strategies concurrently to optimise any national influenza programme.

## Panel Policy considerations for countries wishing to introduce a paediatric influenza vaccination programme

We reassess the cost-effectiveness of the elderly vaccination policy in England because it was one of the first countries to introduce a publicly funded programme of paediatric influenza vaccination. With results suggesting that this is a highly cost-effective health policy, other countries will probably also follow a similar route. We would therefore recommend that decision makers consider the following three questions before adapting and renegotiating their influenza vaccination policies:

1 Are they willing to pay the same per vaccine dose for an elderly influenza vaccination programme in the presence of a national paediatric programme? In our base case scenario in the English context, the mean incremental cost-effectiveness increases from £12 552 per quality-adjusted life-year (QALY) to £29 145 per QALY after a successful preschool-based and school-based programme is introduced with high coverage.
2 How quickly would the vaccines be available and administered via a preschool-based and school-based programme? Our results show that swift paediatric vaccination can negate the requirement for elderly vaccination, whereas slow uptake wastes vaccine doses by protecting children too late to have any effect on the reduction of transmission.
3 Would a focus on the achievement of a high coverage across all paediatric groups be more cost-effective than maintaining a historically low elderly coverage? When combined with previous results, we conclude that the per dose cost-effectiveness of a paediatric (2–16 years) vaccine is higher than that for an elderly vaccine, such that expansion of the paediatric programme as much as possible will always be economically advisable.
